# Clinical comparison of cryoballoon ablation and radiofrequency ablation in the treatment of persistent atrial fibrillation

**DOI:** 10.3389/fcvm.2026.1769845

**Published:** 2026-02-23

**Authors:** Jiaxin Luan, Xinyu Zhang, Zixuan Meng, Jianqing She, Rui Shi, Yixin Zhao, Lihong Fan, Yang Liu, Yue Wu, Hongbing Li

**Affiliations:** 1 Cardiovascular Department, First Affiliated Hospital of Xi'an Jiaotong University, Xi'an, Shaanxi, China; 2 The Ninth Hospital of Xi'an, Xi'an, Shaanxi, China; 3 Xi'an Jiaotong University, Xi'an, Shaanxi, China; 4 Heart Rhythm Center, Royal Brompton and Harefield Hospital, Guys and St Thomas’ NHS Foundation Trust, London, United Kingdom

**Keywords:** cryoballoon catheter ablation, efficacy, persistent atrial fibrillation, radiofrequency ablation, safety

## Abstract

**Aims:**

To analyze and compare the efficacy and safety of cryoballoon catheter ablation (CBA) and radiofrequency ablation (RFA) in the first treatment of patients with persistent atrial fibrillation (PerAF).

**Methods:**

116 patients with PerAF who underwent the first-time catheter ablation were enrolled retrospectively, including 56 patients in CBA group and 60 patients in RFA group. The primary endpoint was symptomatic onset after 3 months of ablation or occurrence of atrial arrhythmia (ATA) with any documented duration more than 30 s. The secondary endpoints were perioperative complications and re-ablation 3 month after procedure.

**Results:**

No remarkable difference existed between CBA group and RFA group in the recurrence rate one year after operation (CBA 16.1% vs. RFA 23.3%, *P* = 0.360). Additionally, the complication rates were similar between the two groups (CBA 25.0% vs. RFA 35.0%, *P* = 0.312).

**Conclusion:**

In this real-world study, the effectiveness and overall safety of CBA and RFA in the treatment of PerAF were comparable.

## Introduction

From the late 20th century to the 21st century, the incidence and prevalence of atrial fibrillation (AF) have shown an increasing trend due to the acceleration of the aging process of the population and the improvement of clinical diagnosis and treatment capabilities (1). AF has gradually become the most common arrhythmia in adults. In 2017, approximately 37.57 million people worldwide were living with AF (2), and about 4.72 million cases with new onset AF were reported in 2019 (3). Currently in China, it is estimated over 4.87 million individuals aged 35 years old and above suffer from AF (4).

Two main methods including medication and intervention are used to control the ventricular rate in clinical practice. Catheter ablation of AF demonstrates significant advantages in terms of efficacy, safety, restoration and maintenance of sinus rhythm, symptom improvement (5), and these advantages arise from the toxic side effects and variable efficacy of pharmacological treatments, as well as the surgical trauma associated with conventional surgical procedures. Consequently, catheter ablation is recommended as the first-line treatment for patients with paroxysmal AF (PAF) who do not respond to or cannot tolerate class I or III antiarrhythmic drugs (AADs) (6).

Currently, catheter ablation techniques primarily include CBA, RFA and the emerging Pulsed Field Ablation. The FIRE AND ICE trial, along with other studies have demonstrated that CBA is not inferior to RFA in terms of efficacy and overall safety for patients with of drug-resistant PAF (7, 8). Subsequent secondary analysis showed that, compared to the RFA group, the CBA group experienced significantly reduced rates of repeat ablation, cardioversion, all-cause rehospitalization and cardiovascular-related rehospitalization during follow-up (9). In a single-center, non-randomized controlled study, 100 patients with persistent AF (PerAF) were evenly divided into the CBA group and the RFA group, and treated them pulmonary vein isolation alone (PVI). It showed that recurrence-free rate of AF was 60% in the CBA group and 56% in the RFA group one year after ablation, indicating that the prognosis without atrial arrhythmia (ATA) was comparable between the two groups (*P* = 0.71) (10). A meta-analysis involving 934 patients from 7 centers showed no significant difference in efficacy or operative complications between CBA and RFA for the treatment of PerAF. CBA reduced the incidence of repeated ablation, but was associated with a higher risk of phrenic nerve paralysis (PNP), whereas RFA increased the risk of pericardial tamponade (11).

Although previous studies have confirmed the efficacy and safety of CBA in PAF, evidence comparing CBA and RFA in PerAF remains limited, particularly in real-world settings. Moreover, how differences in ablation strategies and procedural complexity between energy sources influence clinical outcomes in routine practice is still insufficiently explored. Therefore, this study aimed to compare the efficacy and safety of CBA and RFA for PerAF in a real-world clinical setting.

## Methods

### Study patients

A total of 445 patients admitted to the First Affiliated Hospital of Xi ‘an Jiaotong University from January 1, 2019 to June 30, 2021 with the diagnosis of AF were screened. Patients with PerAF who received the first-time catheter ablation were retrospectively included. The inclusion criteria were: ① ≥18 years old, confirmed as PerAF patients by ECG or 24 h Holter and medical history; ② Evidence of ineffective treatment with at least one AADs (except beta-blockers); ③ Anteroposterior diameter of left atrium ≤60 mm on Echocardiogram; ④ No thrombus in left atrium before the procedure. The exclusion criteria included: ① Any previous history of left atrial ablation or combined with atrial flutter and other ATA; ② Severe valvular heart disease or uncontrolled heart failure; ③ Hemodynamic instability; ④ Combined with pregnancy, untreated hyperthyroidism, malignant tumors (patients with survival less than 1 year); ⑤ Esophageal stenosis, esophageal varices, esophageal cancer, and upper gastrointestinal bleeding; ⑥ Thrombosis in the left atrium; ⑦ Severe kidney disease (CKD stage 5) or allergic to contrast medium; ⑧ Anticoagulant contraindications (severe active bleeding, the source of bleeding should be identified and treated) and related concomitant diseases (such as severe thrombocytopenia) (12, 13). The study was approved by the local ethics committee of the First Affiliated Hospital of Xi'an Jiaotong University.

### Ablation procedure

Before the operation, all patients underwent transesophageal echocardiography to rule out left atrial thrombus and left atrial and pulmonary vein computed tomography (CT) that decided the way of ablation. Anticoagulant therapy was maintained at least 3 weeks before the operation. New oral anticoagulants were discontinued once 24 h before the operation and restarted 6 h post-operation. Warfarin was bridged with low molecular weight heparin at least 3 days before the operation and discontinued 12 h before the procedure. All AADs (except amiodarone) should be discontinued for at least 5 half-lives before operation. During the operation, all patients were under local anesthetic with conscious sedation. Heparin was administered continuously to maintain the activated clotting time (ACT) between 250 and 350s. After ruling out the operation-related contraindications, the patients and their families were fully informed of the risks and benefits of the procedure before signing the informed consent. If vagal response occurred during PVI, ventricular electrode pacing could be applied.

### Cryoballoon ablation procedure

After once successful atrial septum puncture with x-ray guiding, the mapping catheter and the second generation cryoballoon (28 mm, Medtronic Inc.) were advanced into the left atrium to locate and access the target pulmonary vein (PV) ostia. The fitting degree between the balloon and PV was evaluated by angiography after inflating the balloon. Arrhythmogenic PVs were sequentially ablated, with each PV undergoing at least two freeze cycles at temperatures ranging from −30 °C to - 60 °C for 120s −180 s. PV potential was monitored in real time after inflating the cryoballoon until PVI was confirmed. Additional linear ablation was attempted at the left atrial roof and mitral isthmus using cryoballoon. The decision to perform additional linear ablation beyond PVI was left to the operator's discretion, based on intra-procedural assessment of atrial substrate characteristics, rhythm behavior, and clinical judgment. overall complication rate.

### Radiofrequency ablation procedure

After successful atrial septum puncture, Pentaray (Johnson & Johnson)/HDgrid (Abbott) mapping electrode and contact force ablation catheter were introduced into the left atrium to construct the left atrial geometry with the Carto (Johnson & Johnson)/Ensite 3D (Abbott) mapping system. Circumferential ablation was performed and successful bilateral circumferential PV isolation was confirmed. Following PVI, left atrial substrate mapping was conducted, and ablation was performed according to the distribution of low voltage areas. This could include the left atrial anterior wall line, roof line, posterior wall line, mitral valve isthmus, tricuspid valve isthmus, and CFEs ablation.

After all procedure, direct current or AADs cardioversion was conducted to resume sinus rhythm if the patient was still in AF.

### Postoperative management

Patients without acute complications were discharged on day 3 postoperatively with oral anticoagulation for at least 3 months and INR monitoring during oral warfarin therapy. CHA2DS2-VASc and HAS-BLED scores were used to decide whether to continue anticoagulant therapy after 3 months. Oral amiodarone was given to control the cardiac rhythm when the contraindication of amiodarone was excluded, otherwise oral dronedarone or propafenone was given. AADs were maintained at least 3 months. If arrhythmia recurred, individualized antiarrhythmic treatment was formulated according to the patient's 24 h Holter and rhythm maintenance situation. Electrical cardioversion could be performed if persistent ATA occurred within 3 months after operation. Repeat catheter ablation was considered if ATA remained uncontrolled by AADs.

### Follow-up

The first three months post-operation were defined as the blanking period. Early recurrence was defined as an ATA episode lasting ≥30s at follow-up 3 months after catheter ablation, and late recurrence was defined as an ATA episode lasting ≥30s at follow-up 12 months post-ablation.

After discharge, patients were instructed to perform daily pulse self-examination and heart rate monitoring. Regular follow up visits were scheduled at 3 months, 6 months and 1 year after operation to complete 24-hour Holter, transthoracic echocardiography, and other necessary evaluation. Additionally, patients were followed up by telephone at 3-month and 1-year post-operation to inquire about symptoms such as palpitation, chest tightness, shortness of breath, dizziness and other AF-related symptoms, as well as any risks related to treatment such as thromboembolism, hemoptysis, and gastrointestinal bleeding. If the patient could not be contacted, refused to follow up or died unrelated to the procedure during the follow-up, the case was recorded as lost to follow-up. Patients who were lost to follow-up were excluded from subsequent follow-up analyses due to unavailable outcome data.

### Endpoints

The primary endpoint was the occurrence of symptomatic ATA or episodes lasting longer than 30 s after 3 months post ablation. The arrhythmia included atrial tachycardia (AT), atrial flutter (AFL), or AF. The secondary endpoint was perioperative complications, such as atrio-esophageal fistula, PNP, pericardial tamponade, arteriovenous fistula, cerebral embolism, hemorrhage, hematoma. It also included rehospitalization for AF and the need for electrical cardioversion or re-ablation for ATA after the initial operation.

### Statistical analysis

SPSS 25.0 software was used for statistical analysis, comparison of baseline data among groups, clinical features and operation data. The Shapiro - Wilk test was used to determine continuous variables with normal distributions. Continuous data in line with normal distribution and homoscedasticity were presented as mean ± standard deviation and compared by independent sample T-test. Mann–Whitney U test was used to compare the measurement data of non-normal distribution and presented as median and quartile, M (P25, P75). The counting data were expressed as numbers and percentages, being compared by Chi-square test or Fisher exact probability test. Thereinto, Kruskal–Wallis test was used for ordinal rank data. Using propensity score matching (PSM) to balance the confounding factors of the baseline data, and subgroup analyses were performed between the PVI group and the PVI with additional line ablation group to exclude differences in the results due to different ablation methods. *P* < 0.05 was considered as statistical significance.

## Results

### Patients' characteristics

**Figure 1:**
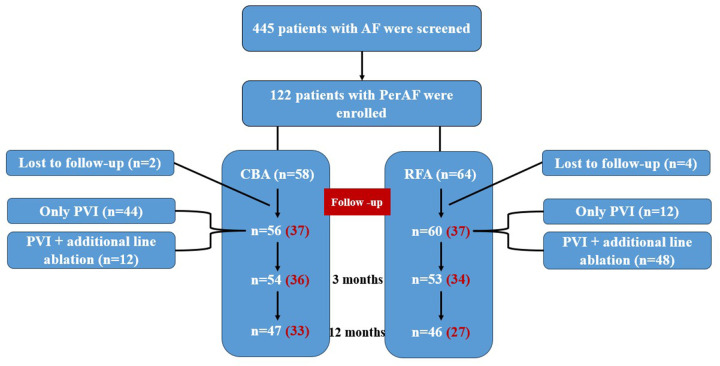
Flow chart of study design. The red number represents the number of subjects after PSM.

A total of 122 eligible PerAF patients were reviewed and recorded, 116 patients were enrolled [64.7% males, mean age: 61.7 ± 10.6 years old, medium duration of AF: 12.0 (1.1, 36.0) months], including 56 patients in the CBA group and 60 in the RFA group. The flow chart of study design was shown in Figure 1.

Patients in CBA group were older [64(57.3,72.0) vs. 62(56.0,65.0) years old, *P* = 0.025] with a higher CHA2DS2-VASc score (*P* = 0.001), while the proportion of males in RFA group was higher (CBA 50% vs. RFA 78.3%, *P* = 0.002), and the difference was statistically significant. In addition, compared with the RFA group, the postoperative creatine kinase (CK) (*P* < 0.001) and creatine kinase-MB (CK-MB) (*P* < 0.001) levels in the CBA group were significant higher in the CBA group (Table 1).

**Table 1:** Comparison of baseline characteristics between CBA and RFA groups.

Variables	CBA (*n* = 56)	RFA (*n* = 60)	*P-value*
Age, years	64 (57.3,72.0)	62 (56.0,65.0)	0.025
Males, *n* (%)	28 (50.0)	47 (78.3)	0.002
BMI, kg/m^2^	24.9 ± 3.2	24.8 ± 2.9	0.930
Smoking, *n* (%)	17 (30.4)	29 (48.3)	0.059
Alcohol, *n* (%)	11 (19.6)	17 (28.3)	0.288
HTN, *n* (%)	32 (57.1)	28 (46.7)	0.272
T2DM, *n* (%)	6 (10.7)	7 (11.7)	1.000
CHD, *n* (%)	8 (14.3)	9 (15.0)	1.000
HF, *n* (%)	2 (3.6)	2 (3.3)	1.000
Stroke/TIA/TE, *n* (%)	6 (10.7)	4 (6.7)	0.656
AF course, months	12 (1.5,36.0)	8.5 (1.0,36.0)	0.807
CHA_2_DS_2_-VASc score			0.001
0	6 (10.7)	17 (28.3)	
1	10 (17.9)	18 (30.0)	
≥2	40 (71.4)	25 (41.7)	
LVEF, %	64.5 (59.3,67.0)	64.5 (57.3,68.0)	0.803
LAD, mm	38.2 ± 4.1	39.6 ± 5.2	0.115
LDL-c, mmol/L	1.7 (1.3,2.3)	1.9 (1.6,2.4)	0.082
HbA1c, %	6.0 (5.6,6.2)	6.0 (5.6,6.1)	0.509
Creatinine, μmol/L	63.0 (56.0,77.0)	60.5 (52.5,69.0)	0.129
NT-pro BNP, pg/mL	818.9 (435.5,1390.0)	608.7 (457.6,1268.3)	0.176
CK, U/	206.0 (167.0,297.0)	117.0 (89.5,148.0)	<0.001
CK-MB, U/L	33.0(25.0,39.0)	18.0(15.3,21.0)	<0.001

AF, atrial fibrillation; BMI, body mass index; CHD, coronary heart disease; CK/CK-MB, creatine kinase (post ablation); HbA1c, hemoglobin A1c; HF, heart failure; HTN, hypertension; LAD, left atrial diameter; LDL-c, low-density lipoprotein cholesterol; LVEF, left ventricular ejection fraction; T2DM, type 2 diabetes mellitus; TE, thromboembolism; TIA, transient ischemic attacks.

### Ablation procedures

467 PVs in 116 PerAF patients were all successfully isolated. In CBA group, PVI alone was performed in 44 cases (78.6%), and PVI with additional line ablation was completed in 12 cases (21.4%). In contrast, in the RFA group, 12 cases underwent PVI alone (20.0%), and 48 cases received PVI with additional line ablation (80.0%). Compared with RFA group, CBA group had a lower proportion of additional line ablation, and the difference was statistically significant (CBA 21.4% *vs*. RFA 80.0%, *P* < 0.001). There were remarkable differences in the application rates of the LA roof line (*P* < 0.001), LA posterior atrial wall line (*P* < 0.001), mitral isthmus line (*P* = 0.001) and tricuspid isthmus line (*P* < 0.001) between the two groups (Table 2).

**Table 2:** The comparison of operation data between CBA group and RFA group.

Variables	CBA (*n* = 56)	RFA (*n* = 60)	*P*
PVI completion, *n* (%)	56 (100.0)	60 (100.0)	ns
PVI, *n* (%)	44 (78.6)	12 (20.0)	ns
PVI+Additional line ablation, *n* (%)	12 (21.4)	48 (80.0)	<0.001
LA Roof, *n* (%)	11 (19.6)	47 (78.3)	<0.001
LA posterior wall line, *n* (%)	0 (0.0)	27 (45.0)	<0.001
LA anterior wall line, *n* (%)	0 (0.0)	1 (1.7)	1.000
Mitral isthmus, *n* (%)	1 (1.8)	14 (23.3)	0.001
Tricuspid isthmus, *n* (%)	0 (0.0)	24 (40.0)	<0.001
CFAEs ablation, *n* (%)	0 (0.0)	2(3.3)	0.496

LA, left atrium.

### Perioperative complications

Three patients (5.0%) in RFA groups experienced severe complications, pericardial perforation/tamponade, and one underwent immediate surgical repair, the other two were treated with pericardial drainage. Two patients (3.6%) developed transient PNP during the operation in CBA group and fully recovered before discharge. Two patients in each group developed acute pulmonary oedema treated with diuretics infusion. (CBA 3.6% *vs*. RFA 3.3%, *P* = 1.000). None of the above patients was life-threatening after active treatment. The incidence of atrial tachyarrhythmia episodes during hospitalization was significantly higher in the RFA group than in the CBA group (CBA 17.9% vs. RFA 35%, *P* = 0.037). However, there was no significant difference in the incidence of AF between the two groups during the blank period. These early rhythm events were not classified as procedural complications. No significant differences in other complications were observed between the two groups.

Overall, there was no significant difference in the incidence of major complications between the two groups (CBA 25.0% vs. RFA 35.0%, *P* = 0.312) (Table 3).

**Table 3:** Comparison of perioperative complications between CBA group and RFA group.

Complications	CBA (*n* = 56)	RFA (*n* = 60)	*P*
Number of complications in an individual			
0	42 (75.0)	39 (65.0)	0.312
≥2	5 (8.9)	1 (1.7)	0.179
Pericardial perforation/tamponade, *n* (%)	0 (0.0)	3 (5.0)	0.267
PNP, *n* (%)	2 (3.6)	0 (0.0)	0.231
Digestive tract symptom, *n* (%)	5 (8.9)	7 (11.7)	0.764
Hemorrhage, *n* (%)	4 (7.1)	0 (0.0)	0.110
Atrio-esophageal fistula, *n* (%)	0 (0.0)	0 (0.0)	ns
Acute left heart failure, *n* (%)	2 (3.6)	2 (3.3)	1.000
Malignant arrhythmia, *n* (%)	1 (1.8)	0(0.0)	0.483

### Primary endpoint during follow-up

**Figure 2:**
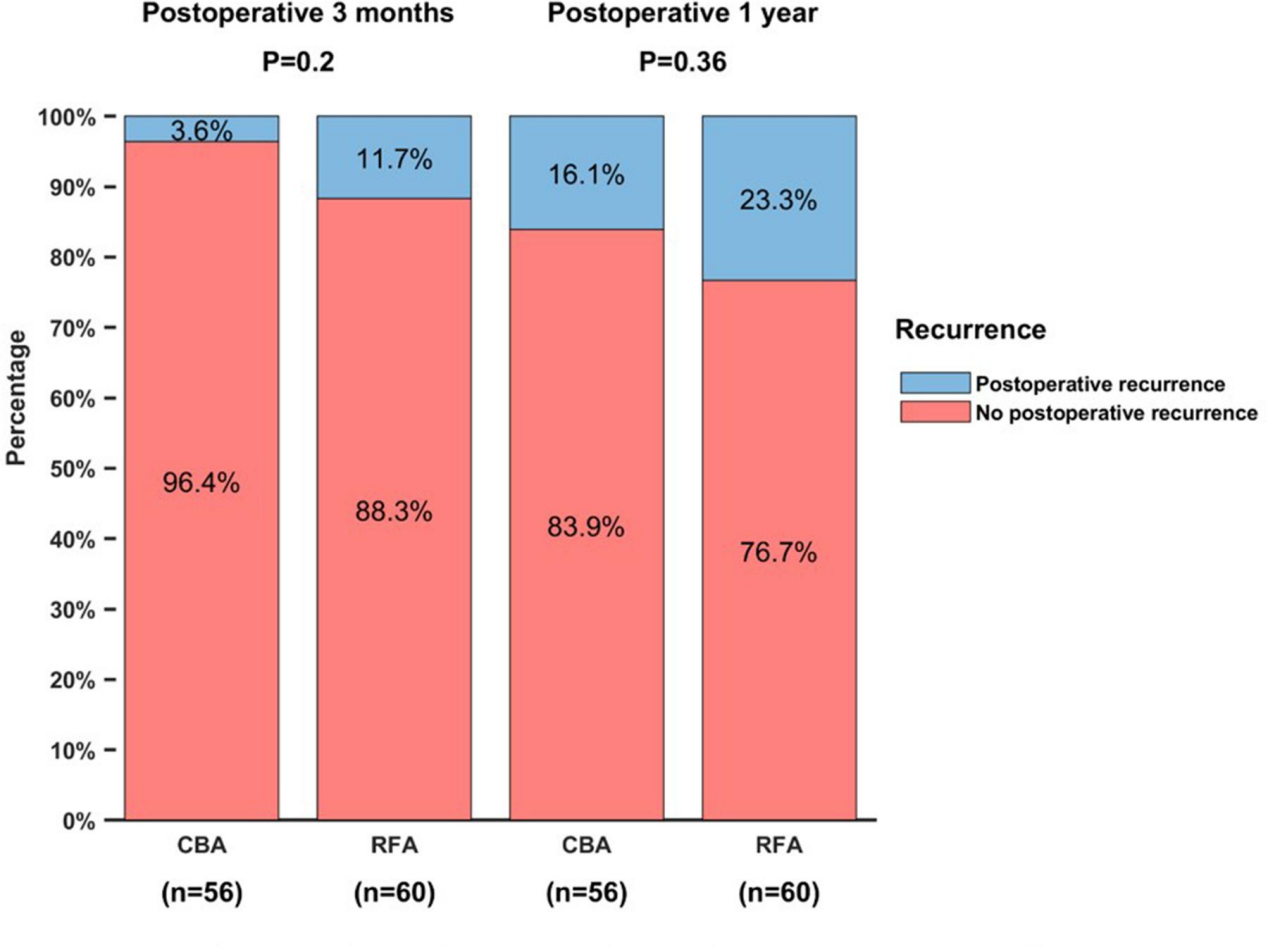
The comparison of recurrence free rate between CBA group and RFA group.

In the CBA group, 9 patients experienced late recurrence during the follow-up and there were 14 patients in the RFA group. The 1-year recurrence-free rate was 83.9% in CBA group and 76.7% in RFA group. There was no significant difference in recurrence-free rate between the two groups (*P* = 0.360) (Figure 2).

116 patients were divided into PVI group and PVI with additional line ablation group, and no significant difference existed in 1-year recurrence rate between the two groups (PVI group 90% vs. PVI with additional line ablation 73.3%, *P* = 0.056). Additionally, CBA group and RFA group were respectively divided into PVI group and PVI with additional line ablation group according to whether additional ablation was performed, showing no remarkable difference in the postoperative 1-year recurrence rate between PVI group and PVI with additional line ablation group in the CBA subgroups and RFA subgroups (CBA group: *P* = 1.000, RFA group: *P* = 0.079).

Due to the statistically significant differences in age, sex and CHA_2_DS_2_-VASc score between CBA and RFA groups. Further Propensity Score Matching (PSM) was performed on 12 baseline data including age, sex, BMI, smoking, drinking, diabetes, hypertension, coronary heart disease, stroke/TIA/TE, AF duration and CHA2DS2-VASc score, 37 pairs of subjects were finally matched (Figure 1). And there was no significant difference in baseline data between CBA group and RFA group after PSM correction (*P* > 0.05) (Table 4). After adjusting the baseline data of the two groups by PSM, binary logistic regression analysis showed that there was no significant difference in 1-year recurrence-free rate between CBA and RFA (89.2% vs. 73.0%, *P* = 0.084) whether CBA or RFA was selected for continuous AF catheter ablation. Further comparative analysis of perioperative complications in the 74 patients revealed that the overall complication rate in the CBA group was not higher than that in the RFA group (CBA 16.2% vs. RFA 35.1%, *P* = 0.109), indicating comparable overall safety between the two groups (Table 5).

**Table 4:** Comparison of no recurrence rate between CBA group and RFA group after PSM.

No recurrence	CBA (*n* = 37)	RFA (*n* = 37)	*P*
3 months postoperation, *n* (%)	36 (97.3)	34 (91.9)	0.327
1-year postoperation, *n* (%)	33 (89.2)	27 (73.0)	0.084

**Table 5:** Comparison of perioperative complications between CBA group and RFA group after PSM.

Complications	CBA (*n* = 37)	RFA (*n* = 37)	*P*
Number of complications in an individual			
0	31 (83.8)	24 (64.9)	0.109
≥2	2 (5.4)	1 (2.7)	1.000
Pericardial perforation/tamponade, *n* (%)	0 (0.0)	1 (2.7)	1.000
PNP, *n* (%)	0 (0.0)	0 (0.0)	ns
Atrio-esophageal fistula, *n* (%)	0 (0.0)	0 (0.0)	ns
Acute left heart failure, *n* (%)	0 (0.0)	0 (0.0)	ns
Digestive tract symptom, *n* (%)	2 (5.4)	6 (16.2)	0.261
Malignant arrhythmia, *n* (%)	0 (0.0)	0 (0.0)	ns
Respiratory tract infection (%)	3 (8.1)	3 (8.1)	1.000
Hemorrhage, *n* (%)	3 (8.1)	0 (0.0)	0.238
Puncture site injury, *n* (%)	0 (0.0)	4(10.8)	0.123

## Discussion

According to the current guideline recommendation, catheter ablation is an established treatment for drug-refractory AF, and RFA is still the main means of AF ablation. In recent years, the CBA as an effective way of ablation, is widely used to treat PAF, its effectiveness and safety has been confirmed by numerous studies (7–9). Compared with PAF, the pathogenesis of PerAF is more complex with more difficult ablation and higher postoperative recurrence rate. About 7.7% of PAF patients will develop PerAF annually (11). Therefore, effective ablation strategies for the treatment of PerAF are still being explored in order to improve prognosis. This study evaluated the efficacy and safety of CBA and RFA in the treatment of PerAF in a real clinical environment, finding that: (1) The efficacy of CBA and RFA in the treatment of PerAF was comparable, postoperative 1-year recurrence rate showed no significant difference; (2) The incidence of perioperative complications in the CBA group was not higher than that in the RFA group, and its overall safety was not inferior to the RFA group; (3) After adjusting for confounding factors by propensity score matching (PSM), there was no significant difference in the 1-year recurrence-free rate between the CBA and RFA groups, and the overall safety of the CBA group was not inferior to that of the RFA group. (4) Whether additional line ablation was performed or not, it did not affect the recurrence-free rate at 1 year after operation. Our findings are consistent with recent randomized trials, including the NO-PERSAF study, which demonstrated comparable efficacy between CBA and RFA in PerAF. Extending these results, our real-world analysis suggests that similar clinical outcomes can be achieved despite substantial differences in ablation strategy complexity between the two energy sources.

### Analysis of efficacy

Among the 116 PerAF patients enrolled in this study, there was no significant difference in the effectiveness of CBA and RFA in treating PerAF (CBA 83.9% *vs.* RFA 76.7%, *P* = 0.360). We divided study population into different subgroups by age, sex and CHA_2_DS_2_-VASc score due to the discrepancy in baseline data, and the 1-year postoperative efficacy of CBA and RFA showed no significant difference among subgroups. To balance the confounding factors, 37 patients were further screened for PSM from CBA and RFA respectively, and the postoperative 1-year recurrence rate was analyzed. The results showed that there was no significant difference in the primary endpoint between the CBA and RFA groups (CBA 89.2% *vs.* RFA 73.0%, *OR* = 3.056, 95%*CI*: 0.861–10.838, *P* = 0.084). The propensity score–matched analysis yielded results consistent in direction with the overall cohort; however, given the sample size of the matched cohort, this analysis should be viewed as supportive rather than confirmatory, and not interpreted as evidence of equivalence between CBA and RFA. Later, we divided 116 patients into recurrence group and sinus rhythm maintenance group 1 year after operation and found no significant association between AF ablation type and postoperative 1-year recurrence rate (*OR* = 0.355, 95% *CI*: 0.065 1.928, *P* = 0.230). In conclusion, this study demonstrated that the efficacy of CBA and RFA treating PerAF was comparable, which was consistent with the previous findings: Atsushi et al. (12) analyzed 253 PerAF patients treated with CBA and 265 PerAF patients treated with RFA, indicating that there was no significant difference in recurrence-free rate between two groups during the follow-up period of 25.5 ± 12.5 months (CBA 72.3% *vs.* 69.8% RFA, adjusted HR 0.85, 95% *CI* 0.59 1.21, *P* = 0.36). In the subgroup analysis of PerAF patients from the FREEZE cohort (13), no difference in primary endpoint was observed between CBA and RFA groups (*P* = 0.95). In a recent randomized controlled study from Norway (14), 101 patients with PerAF were randomized into CBA and RFA groups and followed up for 12 months, the intentionality analysis (ITT) showed no difference in the recurrence free rate between the two groups (CBA 69.2% *vs.* RFA 61.2%, *P* = 0.393), besides, fewer AFL recurrences were recorded in CBA group.

The long-term follow-up results after PVI alone are not satisfactory because of the complexity of the pathogenesis of PerAF, and additional non-PV lesions ablation are often needed (15, 16). The CBA is designed for PVI instead of additional ablation of lesions outside PV. In this study, 12 patients treated with CBA underwent additional line ablation, whereas in the RFA group, more additional ablation procedures were performed in addition to PVI. Therefore, additional demand for ablation could affect the treatment strategy, resulting in a selection bias. To balance this factor, our study divided 116 patients into PVI group and PVI + additional line ablation group, then performed subgroup analysis. It showed that consistent with the results of Atsushi et al., the choice of catheter ablation procedure had no effect on the primary endpoint of treatment for PerAF within subgroups, indicating that the clinical efficacy of CBA treating with PerAF is not limited to PVI alone, but comparable to RFA in the treatment of PVI with additional line ablation (12). However, in the STAR AFII study (17), 589 PerAF patients were randomly assigned at a ratio of 1:4:4 to perform PVI (67 cases), PVI + complex disruptive potential ablation (263 cases), PVI+left atrial roof and mitral isthmus linear ablation (259 cases), respectively. It found that the recurrence rate of AF was not reduced by either complex potential ablation or linear ablation based on PVI (PVI 59% *vs*. PVI with complex potential ablation 49% *vs*. PVI with linear ablation 46%, *P* = 0.15), which was in line with our study. Further analysis in subgroups of CBA and RFA showed that there was no significant difference in 1-year recurrence-free rate between the two groups. The data of João Mesquita et al. on preventive ablation of the tricuspid valve isthmus before AF initial ablation also showed that (18) preventive CTI ablation at the first PVI may not prevent long-term AF recurrence. This result provided data support for CBA treating PerAF with PVI alone. In particular, the widely used second-generation cryoballoon has a more uniform and effective freezing effect, a stronger adaptability to different PV, and a higher fit with PV. While improving the operation process, the therapeutic effect of treating PerAF is gradually recognized, with a sinus rhythm maintenance rate of 60%–80% one year postoperatively and a sinus rhythm maintenance rate of 56% two years postoperatively (19, 20), which is similar to the success rate of RFA in the treatment of PerAF (14). This study also found that the levels of CK and CK-MB after CBA were higher than that of RFA, further reflecting the greater degree of injury during CBA, which was consistent with the previous findings of Michela et al. (21): they compared the influence of open irrigation-tip radiofrequency ablation catheter, cryoablation catheter, visual laser balloon catheter, open-irrigated radiofrequency catheter with direct force measurement on myocardial necrosis after AF ablation, finding the elevated biomarkers of myocardial injury in all patients undergoing PVI, with even higher troponin I (cTnI) and CK-MB values after CBA. They assumed that this might be related to the longer energy transfer time, the larger contact surface between the cryoballoon and the endocardial tissue and the complete occlusion of PV during CBA causing more damage due to reduction of local blood convection heating. However, after an average follow-up of 369 ± 196 days for the patients undergoing these for types of catheter ablation, no significant association was observed between biomarker levels and AF recurrence, that was, higher levels of cardiac biomarkers did not suggest better clinical outcomes, and their physiological significance was still unclear. A study of postoperative injury in MRI (22) also confirmed higher myocardial damage after CBA, it found that compared with RFA, the injured area after CBA was wider, with fewer damage gaps and better continuity. Therefore, these findings suggest that cryoballoon ablation may produce more extensive and continuous lesion formation at the pulmonary vein level, rather than indicating superior clinical effectiveness compared with radiofrequency ablation. Importantly, in our cohort, these biomarker elevations were not associated with an increase in major perioperative complications or other clinically evident adverse outcomes. Also, CBA had lower repeat ablation rate and more persistent PVI. David et al. further highlighted the advantages of CBA PVI by analyzing data from two large prospective registries and found (23) that 11% of patients underwent a second ablation after RFA and 7.8% of patients underwent a second ablation after CBA. Although multiple studies have shown that PVI of CBA was superior to RFA, and CBA is no less effective than RFA in the treatment of PerAF with PVI+additional linear ablation, CBA was expected to construct a larger and more reliable stromal damage areas during linear ablation. However, in linear cryoballoon ablation, the testing of bidirectional block, the improvement of auxiliary wire accuracy, and the establishment of contact safety and stability remained to be solved (11). What's more, the effect of additional PV ablation was still unclear and controversial due to differences in individual identification, lack of uniform ablation endpoint, operation risk and other factors. It has been suggested that the success rate of PerAF ablation can be improved by the identification of left atrial low-voltage region with mapping catheter and matrix modification (24). PVI combined with left atrial appendage isolation has also been shown to improve the success rate of postoperative sinus rhythm maintenance (25, 26). Recent studies have found that ethanol ablation of Marshall vein (VOM) can improve bidirectional mitral isthmus block, and its combination with radiofrequency ablation can further improve the success rate of operation (27, 28). Therefore, ablation strategies for PerAF still need to be continuously explored and improved in order to obtain more effective ablation.

### Analysis of safety

Multiple previous studies have shown that the incidence of complications and overall safety was similar between CBA and RFA regardless of catheter ablation for PAF or PerAF (7, 8, 12, 13). PNP is a characteristic complication of CBA. With the use of the second-generation cryoballoon and the accumulation of previous experience, there were 2 patients (3.6%) in this study developing transient PNP during CBA. Effective preventive measures such as routine phrenic nerve pacing during operation could prevent or reduce the occurrence and aggravation of PNP. Partial studies have reported that RFA has a higher risk of pericardial tamponade than CBA (11, 28). In this study, 3 patients developed pericardial tamponade during RFA, 2 patients received pericardial puncture and drainage, and 1 patient underwent perforation and repair in cardiac operation, all of which were non-life-threatening. However, the slightly higher frequency of complications in the RFA group, despite not reaching statistical significance, suggests a potentially higher procedural risk compared to the CBA group, even though these complications were not life-threatening. The reported overall complication rate included both major and minor events; minor complications such as transient digestive symptoms accounted for a substantial proportion and should be interpreted accordingly when compared with previous studies. Although the overall complication rates were statistically similar, the occurrence of pericardial tamponade exclusively in the RFA group suggests a trend toward more severe mechanical complications compared with CBA, particularly in terms of the level of intervention required. As mentioned earlier, the more complex procedures and additional ablations in the RFA group may have contributed to the higher incidence of complications. It is also important to note that managing these complications required a higher level of intervention, which may not be available in all clinical settings, thereby affecting the generalizability of the safety profile. The incidence of ATA during hospitalization in RFA group was also higher than that in CBA group (CBA 17.9% vs. RFA 35.0%, *P* = 0.037), which may be related to the operation and different energy. RFA adopts thermal injury to achieve the purpose of isolation and bidirectional block, and the lesion damage can spread to the surrounding normal myocardium, aggravating myocardial cell edema and inflammatory reaction, therefore inducing more unstable short-term inner electrical activity and higher incidence of ATA compared to CBA. But there was no significant difference in the recurrence of AF between the two groups during the blank period and the early follow-up period. The incidence of hemorrhage, acute left heart failure, puncture injury, digestive tract reaction, and respiratory tract infection also did not exhibit significant difference between the two groups. The use of perfusion radiofrequency catheter in the past increased the risk of heart failure, whereas the currently improved pressure-sensing radiofrequency catheter remarkably reduced the incidence of heart failure in RFA. Hemorrhagic events were observed exclusively in the CBA group. This finding was descriptive, and no formal analysis of perioperative anticoagulation parameters was performed. Therefore, the potential association with older age and higher CHA2DS2-VASc scores should be regarded as a possible explanation rather than a definitive conclusion. Moreover, the number of ablation lines is indicative of the complexity of the procedure, with more lines potentially leading to a higher risk of complications. This is because each additional line increases the total lesion set within the heart, which can lead to a higher likelihood of damage to adjacent structures, such as the esophagus or the phrenic nerve, or result in a greater inflammatory response. Moreover, a more complex procedure with multiple ablation lines may require longer procedure times, which could increase the risk of complications related to prolonged fluoroscopy exposure or the cumulative effects of anesthesia. While we observed a slightly lower incidence of complications in the CBA group, we were limited in our ability to definitively link the number of ablation lines to complication rates due to the nature of our study design and the limitations imposed by a small sample size. Further research with larger sample sizes and a prospective study design is necessary to better understand the relationship between the number of ablation lines and complication rates.

In conclusion, we assessed and compared the overall safety of CBA and RFA, demonstrating that the incidence of complications in the CBA group was not higher than that in the RFA group. Therefore, this study concludes that the overall safety of CBA is non-inferior to that of RFA. Considering the differences in baseline data, we performed propensity score matching (PSM) and conducted a comparative analysis of perioperative complications in 74 patients, still finding that the safety of CBA was comparable to that of RFA. Our results confirm the safety of CBA.

### Limitations

This study carries inherent limitations due to its retrospective and single-center nature, which may introduce biases that are not present in multi-center, prospective studies. The small sample size of PerAF patients limits the generalizability of our findings and may affect the statistical power to detect significant differences. Additionally, the non-randomized selection of patients could introduce selection biases, potentially skewing the results. The choice of catheter ablation procedure had operator precedence, and the efficacy of the procedure was related to the operators, which could not represent the effect of different centers. Multi-center and prospective studies can be carried out to enroll more patients in order to increase the reliability and applicability of the results later. Secondly, the follow-up period of this study was 1 year, failing to analyze the long-term outcome of catheter ablation. In addition, arrhythmia recurrence assessment relied mainly on scheduled 24-hour Holter monitoring and symptom-based follow-up, which may have underestimated asymptomatic atrial arrhythmia episodes and influenced the reported recurrence rates. We highlight the importance of conducting future research with a larger sample size and a multi-center approach to enhance the statistical power and external validity of the results. This will allow for a more robust evaluation of the long-term recurrence rates and the comparative effectiveness of CBA and RFA. With the application of new monitoring equipment, the reliability and accuracy of subsequent follow-up will be improved. Besides, due to the retrospective nature of our study, we were unable to record the exact procedural durations for each patient in both the CBA and RFA groups. Consequently, we could not provide the average procedure duration for either group, which would have been beneficial in evaluating the comparative efficiency of the two techniques. Moreover, this study did not perform quantitative analysis of patients' symptoms before and after operation, such as EHAR symptom score, and the recurrence rate were unable to provide relevant information to assess the burden of AF, so the efficacy of catheter ablation may be underestimated.

## Conclusion

In this real-world study, the effectiveness and overall safety of CBA and RFA in the treatment of PerAF were comparable.

## Data Availability

The raw data supporting the conclusions of this article will be made available by the authors, without undue reservation.
